# Synthesis and spectral characterization of the first fluorescein-tagged iron(ii) clathrochelates, their supramolecular interactions with globular proteins, and cellular uptake†

**DOI:** 10.1039/d0ra10502c

**Published:** 2021-02-22

**Authors:** Roman O. Selin, Insa Klemt, Viktor Ya. Chernii, Mykhaylo Yu. Losytskyy, Svitlana Chernii, Andrzej Mular, Elzbieta Gumienna-Kontecka, Vladyslava B. Kovalska, Yan Z. Voloshin, Anna V. Vologzhanina, Pavel V. Dorovatovskii, Andriy Mokhir

**Affiliations:** Vernadskii Institute of General and Inorganic Chemistry NASU 32/34 Palladin Prosp. 03080 Kiev Ukraine v.chernii@gmail.com; Organic Chemistry II, Friedrich-Alexander-University of Erlangen-Nuremberg Nikolaus-Fiebiger-Straße 10 91058 Erlangen Germany; Institute of Molecular Biology and Genetics, NASU 150 Zabolotnogo St. 03143 Kyiv Ukraine; Faculty of Chemistry, University of Wroclaw 14 F. Joliot-Curie St. 50-383 Wroclaw Poland; Kurnakov Institute of General and Inorganic Chemistry of the Russian Academy of Sciences 31 Leninsky Prosp. 119991 Moscow Russia; Nesmeyanov Institute of the Organoelement Compounds of the Russian Academy of Sciences 28 Vavilova St. 119991 Moscow Russia; National Research Center Kurchatov Institute 1 Kurchatova Pl. 123098 Moscow Russia

## Abstract

A fluorescein-tagged iron(ii) cage complex was obtained in a moderate total yield using a two-step synthetic procedure starting from its propargylamine-containing clathrochelate precursor. An 11-fold decrease in fluorescence quantum yield is observed in passing from the given fluorescein-based dye to its clathrochelate derivative. An excitation energy transfer from the terminal fluorescent group of the macrobicyclic molecule to its quasiaromatic highly π-conjugated clathrochelate framework can explain this effect. The kinetics of the hydrolysis of the acetyl groups of acetylated fluorescein azide and its clathrochelate derivative in the presence of one equivalent of BSA evidenced no strong supramolecular host–guest interactions between BSA and the tested compounds. Study of a chemical stability of the deacetylated iron(ii) clathrochelate suggested the formation of a supramolecular 1 : 1 BSA–clathrochelate assembly. Moreover, an addition of BSA or HSA to its solution caused the appearance of strong clathrochelate-based ICD outputs. The fluorescence emission anisotropy studies also evidenced the supramolecular binding of the fluorescein-tagged iron(ii) clathrochelate to the BSA macromolecule, leading to a high increase in this type of anisotropy. Subcellular uptake of the fluorescein-tagged molecules was visualized using fluorescence microscopy and showed its distribution to be mainly in the cytosol without entering the nucleus or accumulating in any other organelle. An X-rayed crystal of the above propargylamide macrobicyclic precursor with a reactive terminal C≡C bond contains the clathrochelate molecules of two types, A and B. The encapsulated iron(ii) ion in these molecules is situated in the center of its FeN_6_-coordination polyhedron, the geometry of which is intermediate between a trigonal prism (TP) and a trigonal antiprism (TAP). The Fe–N distances vary from 1.8754(6) to 1.9286(4) Å and the heights *h* of their distorted TP–TAP polyhedra are very similar (2.30 and 2.31 Å); their values of *φ* are equal to 25.3 and 26.6°. In this crystal, the molecules of types A and B participate in different types of hydrogen bonding, giving H-bonded clathrochelate tetramers through their carboxylic and amide groups, respectively; these tetramers are connected to H-bonded chains.

## Abstract 2

The fluorescein-tagged iron(ii) clathrochelate was synthesized for the first time and its accumulation in A2780 cancer cells was studied.

## Graphical abstract

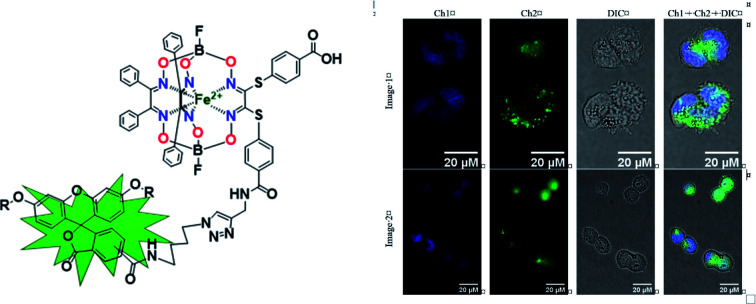

## Introduction

**Fig. 1:**
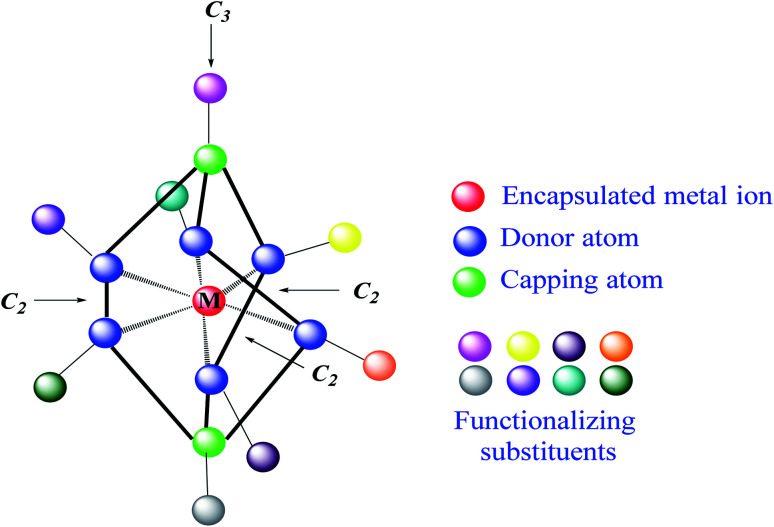
Functionalization of a mononuclear metal-encapsulating clathrochelate framework. The marked symmetry elements appear if the corresponding functionalizing apical and/or ribbed substituents are the same groups.

Clathrochelates^1^ are an unique class of cage metal complexes, with their central metal ion(s) encapsulated in the three-dimensional cavity of an appropriate macropolycyclic organic or organoelement ligand. Designed clathrochelates with terminal reactive group(s) or atom(s) in their molecules possess high flexibility for their easy chemical functionalization in up to four directions, using up to eight sites in the case of mononuclear cage metal complexes (Fig. 1). As it can be seen from Fig. 1, there are six ribbed and two apical potential sites for transformations which can be directly chemically modified using a wide range of organic substituents. This opens a path to different practical applications, including various biological activities, depending on the nature and geometrical positions of these substituents in the cage framework. However, for a long time, the metal complexes of this type were not considered to be prospective biologically active compounds for modern drug therapy because of “classic” requirements^2–4^ for therapeutic prodrugs, despite the presence of a number of exceptions to these rules.^5–7^ In particular, the designed cage metal complexes are reported^8–11^ to be low- and sub-micromolar transcription inhibitors in the transcription systems of T7 RNA and Taq DNA polymerases; they are also able to quench the protein fluorescence of a series of globular proteins.^12^ An *in vitro* study^13^ revealed the high cytotoxicity of an electrophilic iron(ii) hexachloroclathrochelate against the human promyelocytic leukemia cell line; antifibrillogenic activity of the designed carboxyl-terminated iron(ii) mono- and bis-clathrochelates toward insulin and lysozyme has also been observed.^14,15^ Moreover, an ability of the inherently CD-silent iron(ii) clathrochelates to give a strong CD output (induced CD (ICD) signals) in the presence of globular proteins was reported^16,17^ for the first time. The appearance of highly intense clathrochelate-localized ICD spectra for a series of iron(ii) cage complexes as macrobicyclic guests upon their supramolecular binding to bovine and human serum albumins (BSA and HSA) and β-lactoglobulin (BLG) as the hosts suggests their prospective ICD-reporter properties for probing the spatial structure of macromolecules of a wide range of globular proteins and their structural alternations. Regardless of obtaining the above valuable experimental results, the true mechanism of the cytotoxicity and other types of biological activity of these cage complexes remains unknown. Molecular imaging is an efficient prospective method for the detection of the subcellular localization of target molecules.^18,19^ At the same time, a large variety of prospective optical tags (optically active labeling substituents or groups) has been extensively elaborated.^20^ Functionalization of a given clathrochelate molecule with a fluorescent labelling group can be used to monitor its localization in cells; it also allows *in vivo* testing of these cage complexes for a better understanding of their mode of bioactivity. In this paper, we report an efficient synthetic strategy for obtaining the first fluorescein-tagged iron(ii) clathrochelates, whose molecules are functionalized with a terminal fluorophore group, their luminescent properties, and data on their chemical stability in various media, their interaction with globular proteins, and the uptake and localization of these cage metal complexes in cancer cells.

## Experimental

### Materials and apparatus

The reagent tris((1-benzyl-4-triazolyl)methyl)amine (TBTA), CuI, lipase, CALB-L (Novozyme), sorbents, organic bases, and solvents used were obtained commercially. Propargylamide clathrochelate precursor 2 was prepared as described elsewhere.^21^ Diacetyl *N*-(4-azobutyl)-fluorescein-5(6)-carboxamide was obtained using a known synthetic procedure.^22^ Bovine Serum Albumin (BSA) and human serum albumin (HAS) were obtained commercially (SAF® and Fisher Bioreagents, respectively). *β*-Lactoglobulin (BLG), lysozyme (LYZ), human insulin, trypsin, and analytical grade DMSO were also purchased from SAF®. 50 mM Tris–HCl aqueous buffer with pH 7.9 was used as the solvent for all optical studies. All these experiments were performed in a standard 1 × 1 cm quartz cell.

Analytical data (C, H, N contents) were obtained with a Carlo Erba model 1106 microanalyzer. High-resolution APPI mass spectra were obtained using a maXis 4G UHR TOF mass spectrometer (Bruker Daltonik).

^1^H and ^13^C{^1^H} NMR spectra of the complexes under study were recorded from their solutions in acetone-*d*_6_ and DMSO-*d*_6_ with a Bruker Avance 400 spectrometer. The ^1^H and ^13^C (^1^H) NMR measurements were obtained using the residual signals of these solvents.

CD spectra were recorded on a Jasco J-1500CD spectrometer in the range 300–600 nm in 0.1 nm steps with a scan of 3001 points; three scans were averaged for each of the ICD spectra in the visible and near UV ranges. For the latter measurements, stock 5 mM solutions of compounds 1, 3, 4, and 5 in DMSO and 0.2 mM solutions of BSA, HSA, BLG, and LYZ in 50 mM Tris–HCl aqueous buffer with pH 7.9 were prepared. Mixtures of 0.8 mL of this buffer, 0.2 mL of a protein solution, and 4 μL of the stock solution of a given compound were used for the fluorescence experiments. Fluorescence excitation, emission, and anisotropy spectra were measured using a Cary Eclipse fluorescence spectrophotometer (Varian, Australia); the coupled UV-vis experiments were performed with a Shimadzu UV-3600 UV-vis-NIR spectrophotometer. Quantum yields for compounds 4 and 5 were obtained in 50 mM Tris–HCl aqueous buffer with pH 7.9 using a solution of fluorescein in ethanol as the reference fluorophoric standard (its quantum yield *φ*_FL_ is equal to 0.97). For this purpose, the DMSO stock solutions of clathrochelate 4 and modified fluorescein 5 were diluted in 50 mM Tris–HCl aqueous buffer with pH 7.9 to concentrations possessing equal optical densities at 472 and 490 nm, respectively, to that of fluorescein; due to the dilution in the above buffer, the quantity of DMSO in the final solutions was negligibly small. Fluorescence of all the obtained solutions was excited at the corresponding wavelengths (472 nm for complex 4 and fluorescein and 490 nm for compound 5 and fluorescein) and the area below each of these spectra (*S*_dye_ for 4 and 5 and *S*_FL_ for fluorescein as reference) was calculated. Then, the fluorescence quantum yields *φ*_dye_ for these compounds were calculated using the following equation: *φ*_dye_ = *φ*_FL_ × (*S*_dye_/*S*_FL_) × (*n*_H_2_O_/*n*_EtOH_)^2^, where *n*_H_2_O_ and *n*_EtOH_ are the refractive indexes of water and ethanol, respectively.

### Study of uptake and subcellular localization of iron(ii) clathrochelate with fluorescent labelling group

Human ovarian cancer A2780 cells (Merck, 93112519), selected as a representative cancer cell line, were cultured according to the recommendations of Deutsche Sammlung von Mikroorganismen und Zellkulturen GmbH (DSMZ) in Roswell Park Memorial Institute (RPMI) 1640 medium supplemented with 10% FBS (fetal bovine serum), 1% l-glutamine and 1% penicillin/streptomycin (all SAF®). One day before the experiment, A2780 cells were seeded at a concentration of 80 cells per μL in 500 μL of culture medium on a 35 mm-diameter imaging dish (μ-Dish, high, SAF® GmbH, Germany) and incubated overnight. The next day, the cells were washed two times with Dulbecco's Phosphate-Buffered Saline (DPBS) and incubated for 4 h with 9 μM of complex 4 in growth medium containing 5% FBS, 1% l-glutamine, 1% penicillin/streptomycin, and 1% DMSO. Subsequently, the cells were washed two times with DPBS, then 4 ng mL^−1^ Hoechst 33342 in culture medium was added and incubated for 30 min. The cells were washed again two times with DPBS and images were obtained using a Zeiss Axio Vert.A1 equipped with two filter sets (excitation/emission 365/445(50) nm for the 49 DAPI channel and excitation/emission 470(40)/525(50) nm for the 38 blue channel) using the objective 40×/1.30 and an oil DIC.

### Synthesis, analysis and spectral characteristics of the fluorescein-tagged iron(ii) clathrochelates

**Fig. 2:**
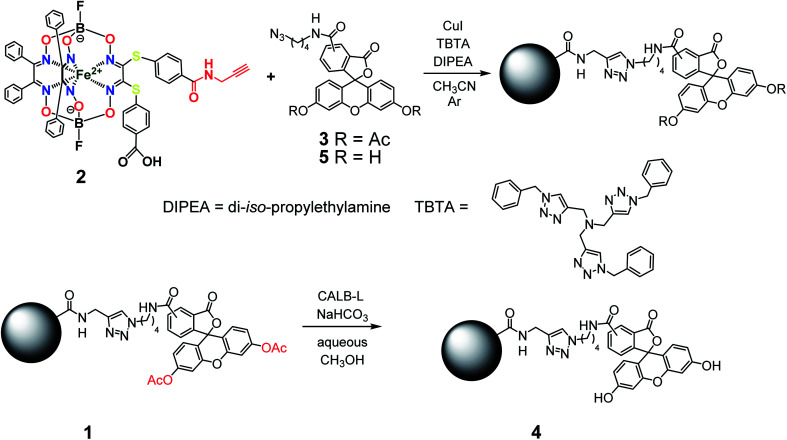
Elaborated synthetic pathway to obtain a given monoribbed-functionalized iron(ii) clathrochelate molecule containing a single terminal fluorescent group in one of its ribbed substituents.

The general synthetic pathway to obtain the new cage iron(ii) complexes is presented in Fig. 2.

#### Complex 1

Clathrochelate precursor 2 (15 mg, 14.7 μmol), fluorescein azide 3 (8.2 mg, 14.7 μmol), CuI (0.3 mg, 1.47 μmol) and TBTA (0.78 mg, 1.47 μmol) were dissolved in acetonitrile (0.6 mL) under argon, then di-iso-propylethylamine (12.8 μL, 73.57 μmol) was added. The reaction mixture was stirred for 4 h at 60 °C and then 1 M aqueous hydrochloric acid (1 mL) was added. The obtained solution was extracted with dichloromethane (3 mL in three portions). The combined extract was washed with a brine solution, dried with Na_2_SO_4_ and separated using column chromatography on silica gel (eluent: ethyl acetate–methanol 7 : 3 mixture), giving the red solid target product with *R*_f_ = 0.28 after evaporation of the corresponding major elute. Yield: 7.8 mg (5.0 μmol, 34%). Calc. for C_76_H_57_B_2_F_2_FeN_11_O_17_S_2_: C, 57.90; H, 3.65; N, 9.78; S, 4.07. Found (%): C, 57.59; H, 3.69; N, 9.55; S, 3.99. MS APPI (positive range) *m*/*z*: Calcd. for C_76_H_57_B_2_F_2_FeN_11_O_17_S_2_: 1575.29, found: 1576.2944 [M + H^+^]^+^. ^1^H NMR (400 MHz, acetone-*d*_6_) *δ* 0.97 (d, *J*_^1^H–^1^H_ = 6.1 Hz, 6H, C*H*_3_), 1.54 (*p*, *J* = 7.1 Hz, 2H, C*H*_2_), 1.86 (q, *J*_^1^H–^1^H_ = 7.3 Hz, 2H, C*H*_2_), 3.36 (dt, *J*_^1^H–^1^H_ = 7.1, 3.7 Hz, 2H, C*H*_2_), 4.32 (t, *J*_^1^H–^1^H_ = 7.0 Hz, 2H, C*H*_2_), 4.48 (d, *J*_^1^H–^1^H_ = 4.9 Hz, 2H, C*H*_2_), 6.86–6.75 (m, 4H, 2*H*–Ar + *H*–N), 7.10–7.04 (m, 2H, *H*–Ar), 7.12 (d, *J*_^1^H–^1^H_ = 8.3 Hz, 2H, *H*–Ar), 7.31–7.15 (m, 23H, *H*–Ar), 7.37–7.31 (m, 1H, *H*–Ar), 7.75–7.67 (m, 2H, *H*–Ar), 7.82–7.74 (m, 2H, *H*–Ar), 8.11 (s, 1H, *H*–Ar), 8.18 (dd, *J* = 8.0, 1.6 Hz, 1H, *H*–Ar), 8.39–8.33 (m, 1H, *H*–Ar). ^13^C{^1^H} NMR (101 MHz, acetone-*d*_6_) *δ* 20.10, 24.87, 26.37, 27.56, 28.38, 28.57, 28.76, 28.96, 29.15, 29.34, 29.53, 29.70, 39.02, 49.39, 81.33, 110.47, 116.23, 118.40, 123.54, 124.34, 126.61, 128.00, 128.03, 128.30, 129.11, 129.18, 129.28, 130.31, 130.61, 134.70, 135.27, 151.41, 152.70, 154.76, 157.70, 167.78, 168.45, 205.35.

#### Complex 4

Complex 1 (10 mg, 6.34 μmol) was suspended in methanol (1 mL), then NaHCO_3_ aqueous solution (25%, 1 mL) and lipase CALB-L (0.1 mL) were added. The reaction mixture was stirred for 48 h at 40 °C, then the solid product was filtered off and recrystallized from methanol. Yield: 6 mg (4.02 μmol, 63%). MS APPI (positive range) *m*/*z*: Calcd. for C_72_H_53_B_2_F_2_FeN_11_O_15_S_2_: 1491.27. Found: 1492.2752 [M + H^+^]^+^. ^1^H NMR (400 MHz, DMSO-*d*_6_) *δ* 1.48 (dt, *J*_^1^H–^1^H_ = 42.7 and 7.7 Hz, 4H, CH_2_), 1.98–1.66 (m, 4H, CH_2_), 3.41–3.16 (m, 4H, C*H*_2_), 4.35 (dt, *J*_^1^H–^1^H_ = 29.1 and 7.0 Hz, 4H, C*H*_2_), 4.50 (dd, *J*_^1^H–^1^H_ = 11.2 and 5.4 Hz, 4H, C*H*_2_), 6.57 (tq, *J*_^1^H–^1^H_ = 6.0, 3.7 and 2.8 Hz, 10H, *H*–Ar), 6.70 (t, *J*_^1^H–^1^H_ = 2.4 Hz, 4H, *H*–N), 7.29 (d, *J*_^1^H–^1^H_ = 8.1 Hz, 8H, *H*–Ar), 7.38 (d, *J*_^1^H–^1^H_ = 5.3 Hz, 40H, *H*–Ar), 7.67 (s, 1H, *H*–Ar), 7.92–7.82 (m, 8H, *H*–Ar), 7.95 (d, *J*_^1^H–^1^H_ = 2.5 Hz, 2H, *H*–Ar), 8.00 (d, *J*_^1^H–^1^H_ = 2.4 Hz, 1H, *H*–Ar), 8.07 (d, *J*_^1^H–^1^H_ = 8.0 Hz, 1H, *H*–Ar), 8.16 (d, *J*_^1^H–^1^H_ = 8.1 Hz, 1H, *H*–Ar), 8.23 (d, *J*_^1^H–^1^H_ = 8.2 Hz, 1H, *H*–Ar), 8.45 (s, 1H, *H*–Ar), 8.70 (t, *J*_^1^H–^1^H_ = 5.6 Hz, 1H, *H*–Ar), 8.85 (t, *J*_^1^H–^1^H_ = 5.6 Hz, 1H, *H*–Ar), 9.07 (dd, *J*_^1^H–^1^H_ = 11.4 and 6.1 Hz, 2H, *H*–Ar), 10.16 (d, *J*_^1^H–^1^H_ = 2.6 Hz, 4H, *H*–Ar), 13.11 (s, 2H, *H*OOC).

### Single crystal X-ray diffraction experiment

A single crystal of the complex 2·0.75C_7_H_16_ suitable for the synchrotron X-ray diffraction experiment was grown from a chloroform–heptane 1 : 5 mixture. Intensities of the reflections for this crystal were collected with the K4.4 Belok beamline of the Kurchatov Synchrotron Radiation Source (NRC Kurchatov Institute, Moscow, Russia) at the wavelength of 0.7927 Å using a Rayonix CCD 165 detector. Data collection was performed at the low temperature of 100.0(2) K using an Oxford CryoJet (Oxford Cryosystems). Image integration was performed using iMosflm software.^23^ The integrated intensities were empirically corrected for absorption using the Scala program.^24^ Crystal data for 2·0.75C_7_H_16_ at 100.0(2) K: C_52.5_H_45.5_B_2_F_2_FeN_7_O_9_S_2_*M* = 1098.05, triclinic, space group *P*1̄, *a* = 13.293(3) Å, *b* = 18.175(4) Å, *c* = 22.565(5) Å, *α* = 104.83(3)°, *β* = 103.52(3)°, *γ* = 90.14(3)°, *V* = 5113(2) Å^3^, *Z* = 4, *D*_calc_ = 1.427 g cm^−3^, *μ* = 0.579 mm^−1^, 17347 independent reflections (*R*_int_ = 0.241), 7195 observed reflections, final convergence factors *R*_1_[*I* > 2*σ*(*I*)] = 0.096, w*R*(*F*^2^) = 0.227 and GOF = 0.992. The structure was solved by the direct method and refined by full-matrix least squares against *F*^2^. Non-hydrogen atoms were refined anisotropically; the positions of hydrogen atoms were calculated and all hydrogen atoms were included in the refinement by the riding model using *U*_iso_(H) = 1.5*U*_eq_(X) for methyl groups and 1.2*U*_eq_(X) for the other atoms. The crystal structure was refined as a twin using HKLF 5 refinement. All calculations were made using the SHELXL2014 (ref. 25) and OLEX2 (ref. 26) program packages. CCDC 2041069 contains the ESI† crystallographic data for this paper.

## Results and discussion

### Synthetic strategy towards the target tagged iron(ii) cage complexes and their preparation

**Fig. 3:**
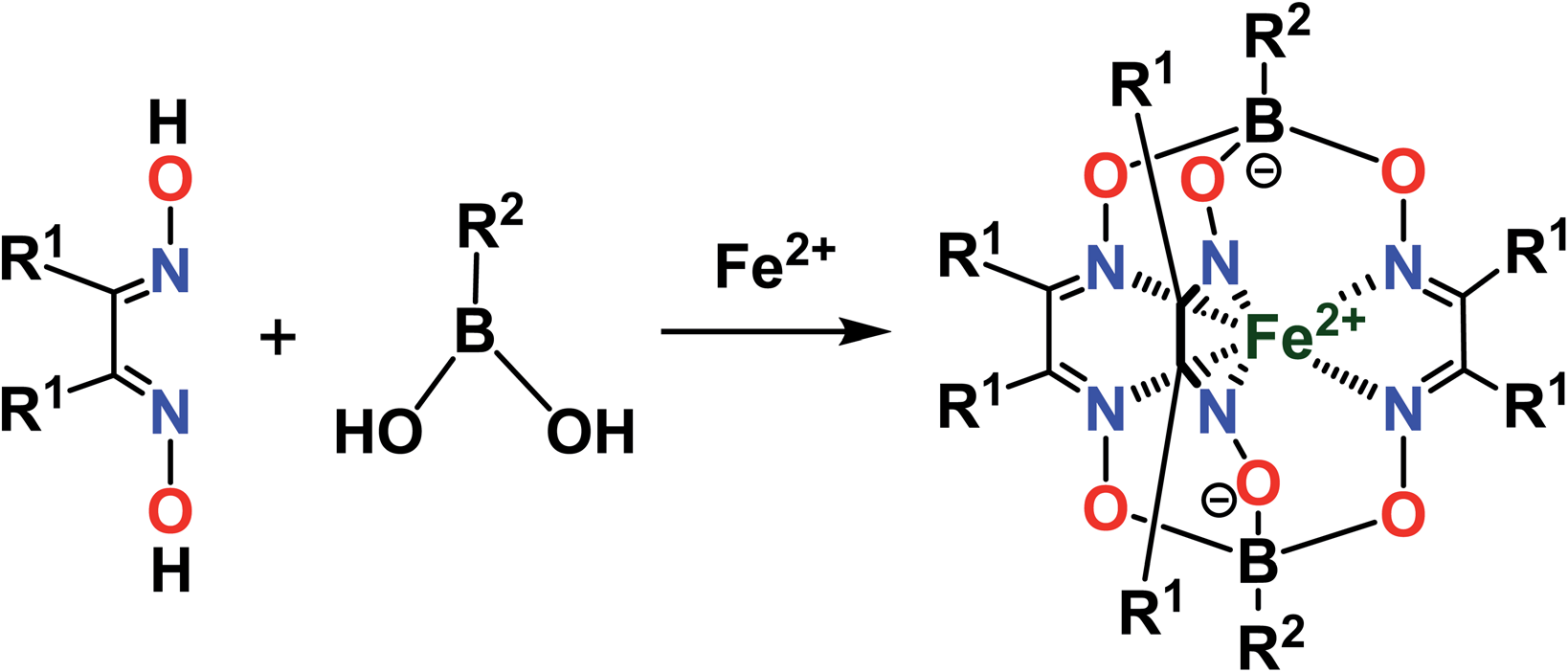
Template synthesis of the boron-capped iron(ii) clathrochelates with equivalent apical cross-linking and ribbed chelating fragments.

The designed clathrochelate tris-α-dioximate molecules with reactive inherent or terminal groups or atoms are quasiaromatic macropolycyclic systems and, therefore, can be easily functionalized using the classical methods of modern organic chemistry.^1^ Very few synthetic approaches for obtaining metal clathrochelates of this type have been developed to date. Despite the metal-promoted C–C cross- and homo-coupling reactions of the suitable halogenoclathrochelate precursors that have been recently developed,^9,10,27–29^ only three main synthetic approaches have been successfully used to date for effective preparation in a reasonable yield. The first of them is based on template condensation on a suitable metal ion as a matrix^30^ (Fig. 3) with a statistical mixture of a given α-dioxime and an appropriate Lewis acid (in particular, a suitable boronic acid or its ester). This method, based on direct *one-pot* condensation, is reported^1,30^ to be the most widely used for the synthesis of a number of cage metal complexes of this type, the molecules of which contain equivalent apical cross-linking and ribbed chelating fragments in their encapsulating macropolycyclic ligands. Unfortunately, its use is substantially limited or even prohibited in the case of the functionalized α-dioximate ligand's synthons, the additional donor group(s) of which are able to form strong coordination bond(s) with a templating metal ion, thus competing with the two N-donor oxime groups of this synthon and causing a decrease in the yield of the target clathrochelate complex. Moreover, such uncoordinated oxime groups are highly reactive towards various ketones, aldehydes, unsaturated bonds, *etc.*^31^ As a result, the triribbed-functionalized metal clathrochelates with equivalent chelate and apical fragments have been prepared mainly by exploring the reactivity of their hexahalogenoclathrochelate precursors (including in metal-catalyzed (promoted) processes) in various nucleophilic substitution and cross-coupling reactions.^1,30^

**Fig. 4:**
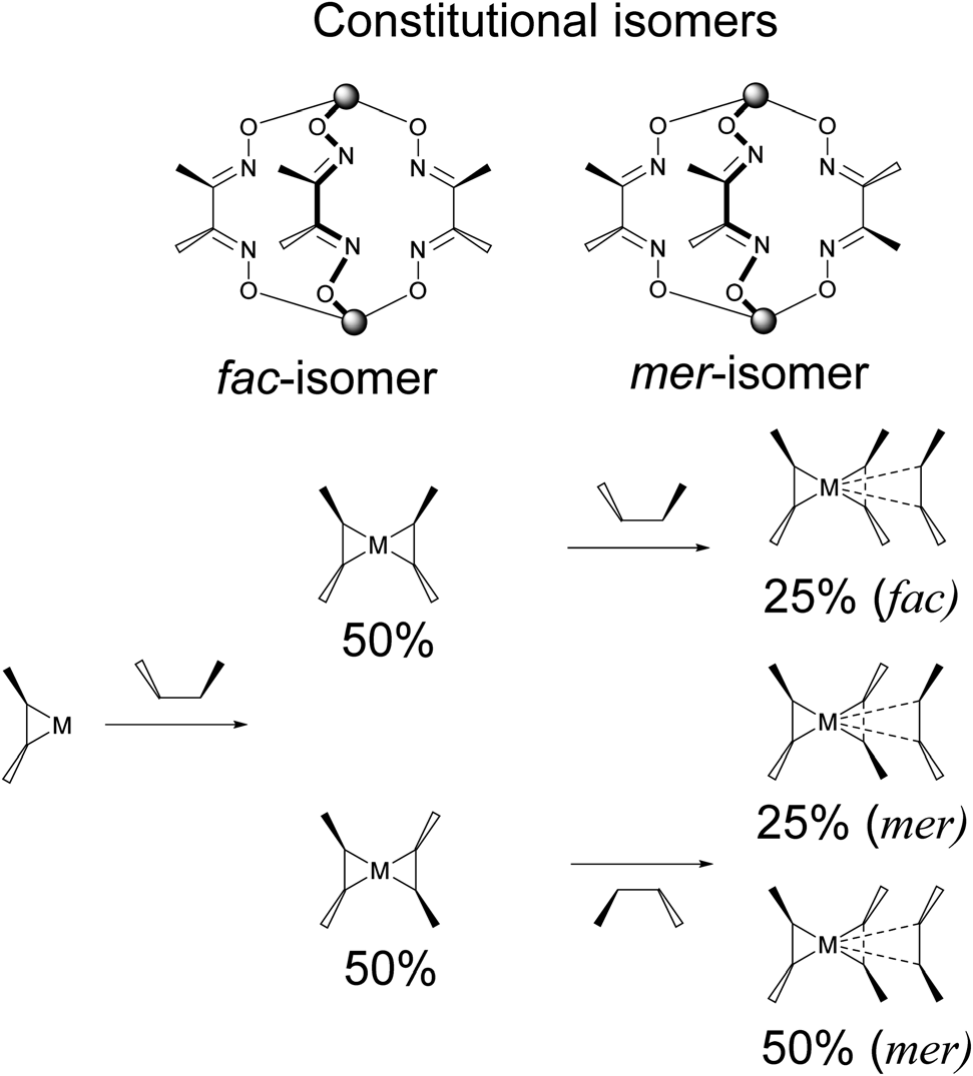
Statistical formation of the constitutional *fac*- and *mer*- isomers of a clathrochelate complex-the derivative of a chelating ligand's synthon with non-equivalent substituents.

From a theoretical point of view, the above direct method may be also used for the synthesis of cage complexes-the derivatives of macropolycyclic ligands with non-equivalent apical and ribbed fragments. Unfortunately, the above template condensations mainly caused the predominant formation of the corresponding symmetric cage molecules; these clathrochelate compounds typically possess a low solubility in the solvents used to perform such *one-pot* condensations. Their precipitation resulted in a shift of equilibrium in these reaction mixtures, thus leading to a formation of the abovementioned symmetric macrobicyclic by-products. Moreover, in many cases, it is very hard or even impossible to separate a mixture of the formed target and side clathrochelate products, especially if the former complexes are minor products or are formed only in trace amounts. In the case of the cage metal complexes-the derivatives of an α-dioximate ligand's synthon with non-equivalent substituents-the direct template reaction gives a mixture of two constitutional *fac*- and *mer*-isomers of the target clathrochelate complex, as shown in Fig. 4.^1,30^ As a result, the statistical synthetic approach has been successfully implemented^32,33^ only for preparation in a low total yield of iron(ii) clathrochelates with non-equivalent capping groups using a mixture of the appropriate boronic acids.

**Fig. 5:**
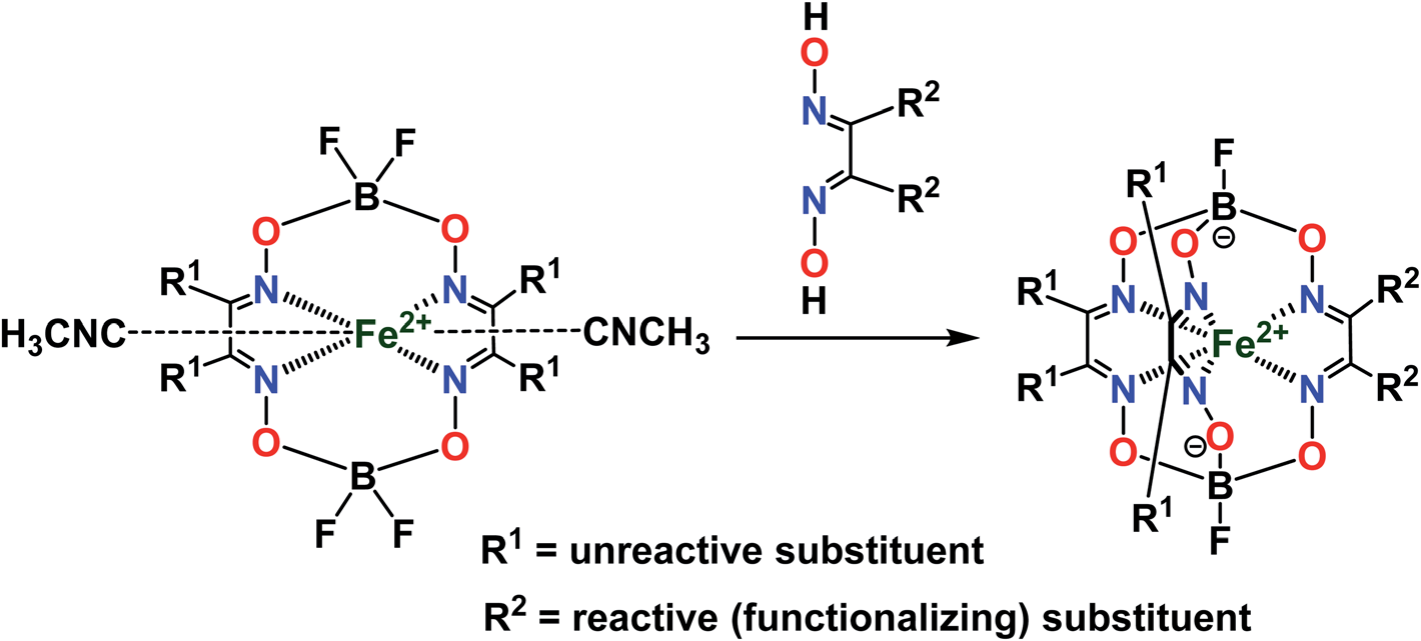
General synthetic approach for preparation of the monoribbed-functionalized iron(ii) clathrochelates.

To avoid the formation of side products, the template condensation (cycloaddition) reactions using the initially obtained BF2-cross-linked bis-α-dioximate metal(ii) complexes as the macrocyclic precursors with halogeno-α-dioximes have been elaborated (Fig. 5).^34–36^ Unfortunately, there are very limited examples of chemically stable macrocyclic complexes of the former type, which do not undergo side symmetrization reactions giving very thermodynamically stable symmetric tris-dioximate clathrochelate by-products, and which, therefore, can be used for such a condensation. To date, these cycloaddition reactions have been exclusively implemented for the macrocyclic bis-dioximate iron- and cobalt(ii)-containing derivatives of α-benzildioxime as an aromatic, structurally rigid and chemically robust ligand's synthon.

The third synthetic method to obtain the target cage complexes with non-equivalent ribbed chelate fragments is based on a stepwise nucleophilic substitution of their reactive halogenoclathrochelate precursors with various *S*,*N*,*O*,*S*,*P*-nucleophiles.^1,30,37–42^ The nature of the clathrochelate products of these reactions is described in literature^1,30,38,41^ as affected not only by the nature of a given nucleophilic agent but also those of the solvent used and of the reaction conditions.

**Fig. 6:**
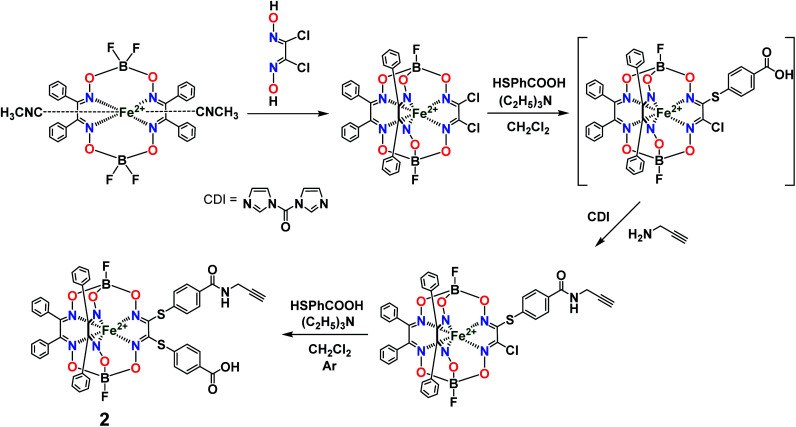
General synthetic pathway to a monocarboxylomonopropargylamine iron(ii)-encapsulating clathrochelate precursor.

Previously, we obtained^21^ the propargylamide iron(ii) cage complex 2 as a prospective clathrochelate precursor for the metal-promoted reactions, the molecule of which contains a terminal C≡C bond. Complex 2 was synthesized using a combination of template condensation and stepwise nucleophilic substitution with an *in situ* chemical transformation of the terminal carboxyl group of its macrobicyclic intermediate (Fig. 6). From a general point of view, it is possible to obtain the corresponding difunctionalized iron(ii) clathrochelate with two terminal carboxyl groups and then implement its further modification with a propargylamine group. However, the order of chemical transformations of a given dichloroclathrochelate precursor shown in Fig. 6 gave the target macrobicyclic complex 2 in an higher overall yield.

**Fig. 7:**
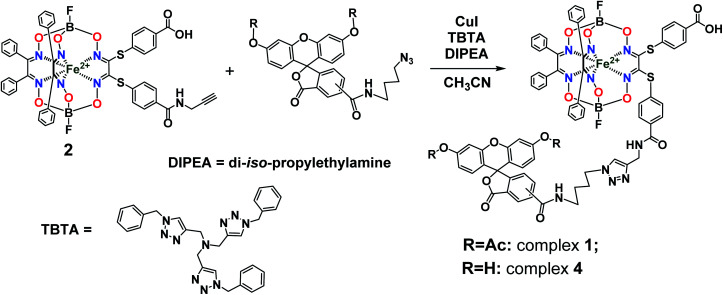
Functionalization of an iron(ii)-encapsulating clathrochelate precursor with the single terminal fluorescein group.

**Fig. 8:**
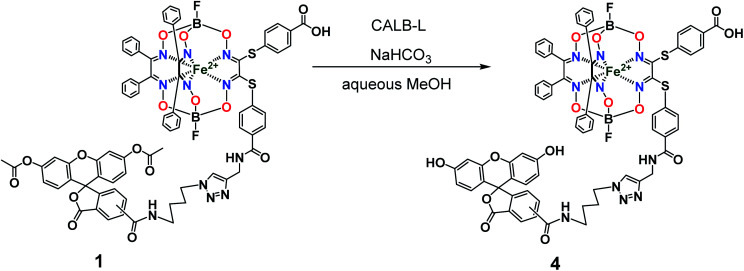
Chemical transformation of an iron(ii) clathrochelate molecule with the single acylated fluorescein group giving its deprotected macrobicyclic derivative.

So, the fluorescein-functionalized iron(ii) cage complex 4 with a terminal fluorophoric group was obtained from its propargylamide-containing clathrochelate precursor 2 using a two-step synthetic procedure shown in Fig. 7. During our preliminary experiments, we tried to perform a direct reaction of this macrobicyclic precursor with the appropriate fluorescein-containing azide component. However, we failed to obtain the target clathrochelate product 4 in a reasonable yield, if any, using different reaction conditions (probably because the presence of hydroxyl groups in this fluorescein-based molecule prevents a formation of the corresponding intermediate copper complex, thus inhibiting the target copper-catalyzed “click”-reaction). Therefore, we initially performed acetylation of the hydroxyl groups and the corresponding acetylated fluorescein-containing clathrochelate intermediate 1 was obtained in a moderate yield of approximately 30%. The formation of substantial amounts of by-products in this 1,3-dipolar cycloaddition could be caused by partial hydrolysis of the acetyl groups of its fluorescein-containing azide component under the reaction conditions used. We tested various synthetic approaches to deprotect the acetylated hydroxyl groups of 1 to obtain the target cage complex 4. Their partial hydrolysis, which easily proceeds under these reaction conditions, also causes a competing parallel process of the complete destruction of 1. Indeed, its clathrochelate tris-dioximate framework is chemically unstable under the “classical” aqueous basic conditions of a hydrolysis reaction of these groups and undergoes complete destruction with elimination of the functionalizing fluorescein group. Such a side process can be easily detected by naked eye as it causes the appearance of the characteristic green coloration in aqueous basic media. To prevent a complete destruction of the cage framework of 1, we used, for the first time, enzymatic hydrolysis with lipase CALB-L as a catalyst, thus giving the target deacetylated clathrochelate 4 in almost quantitative yield (Fig. 8).

**Fig. 9:**
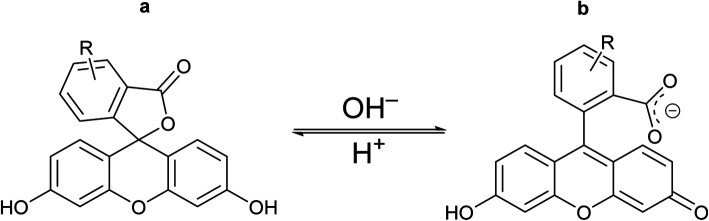
Chemical drawings of the closed (a) and open (b) tautomeric forms of the fluorescein molecule.

The obtained complexes were characterized using elemental analysis, HR-APPI mass spectrometry; UV-vis, fluorescence, and ^1^H and ^13^C{^1^H} NMR spectra; and single-crystal X-ray diffraction (for clathrochelate precursor 2).

### Single crystal X-ray structure of a monocarboxyl-containing clathrochelate precursor with the single terminal reactive C≡C group

**Fig. 10:**
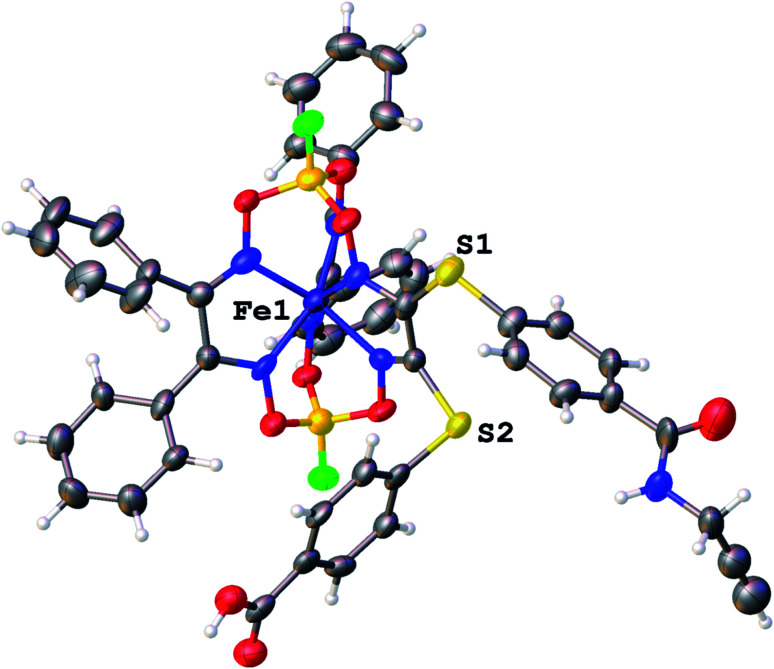
General view of an independent molecule of 2 (type A) in a representation of its atoms as thermal ellipsoids (*p* = 50%).

**Fig. 11:**
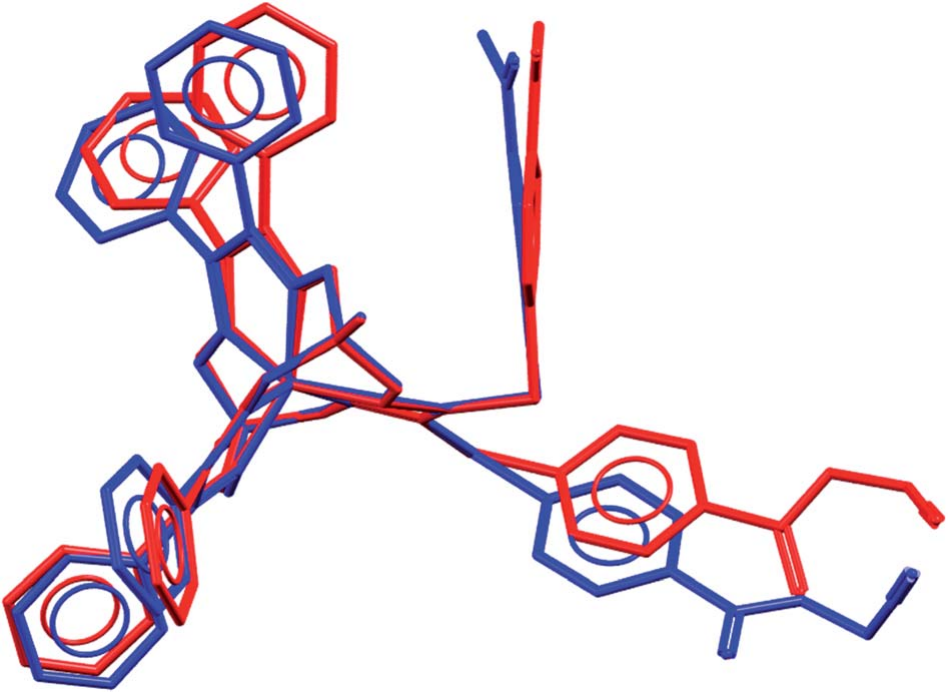
Comparison of the molecular conformations of two independent molecules of 2 (types A and B); views along their B⋯Fe⋯B pseudoaxes.

**Fig. 12:**
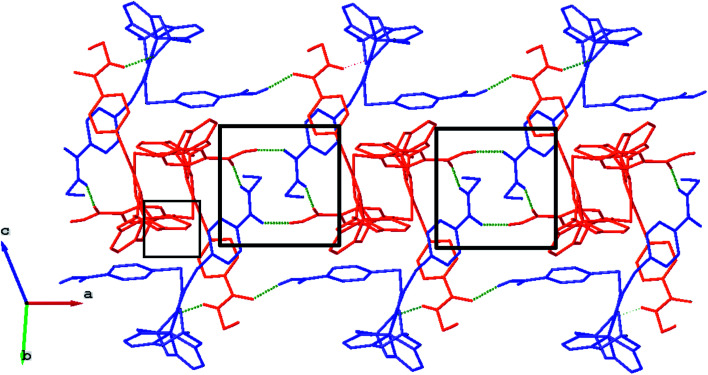
Fragment of the H-bonded clathrochelate chains in the X-rayed crystal 2·0.75C_7_H_16_. The clathrochelate molecules of types A and B are shown in red and blue colors, respectively; solvent molecules, phenyl rings and all H(C) atoms are omitted for clarity. H-bonded clathrochelate tetramers, which are formed within the above chains, are marked using the black squares.

We failed to grow single crystals of the new fluorescein-containing iron(ii) cage complexes, but unexpectedly succeeded in a growing those of their clathrochelate precursor 2 which were suitable for the synchrotron X-ray diffraction (XRD) experiment. The X-rayed crystal of 2·0.75C_7_H_16_ contains the clathrochelate molecules of types A and B; a general view of one of these molecules is shown in Fig. 10, while the main geometrical parameters of their cage frameworks, as well as those of analogous fluoroboron-capped monoribbed-functionalized arylsulfide iron(ii) clathrochelates with known XRD structures, are listed in Table 1. The encapsulated iron(ii) ion in all these cage molecules is situated in the centre of its FeN_6_-coordination polyhedron, the geometry of which is intermediate between a trigonal prism (TP, distortion angle *φ* = 0°) and a trigonal antiprism (TAP, *φ* = 60°). Fe–N distances in the macrobicyclic molecules 2 of types A and B vary from 1.8754(6) to 1.9286(4) Å and the heights *h* of their distorted TP–TAP polyhedra are very similar, equal to 2.30 and 2.31 Å, respectively. The values of their bite (chelate N–Fe–N) angles *α* are characteristic of the boron-capped aromatic α-dioximate iron(ii) clathrochelates,^1,30^ while their values of *φ* are equal to 25.3 and 26.6°, respectively. A comparison of the general views of A and B is displayed in Fig. 11. Free rotation along their ordinary C–S bonds without any steric clashes is allowed and their conformations are different mainly in an orientations of the arylsulfide ribbed substituents at a cage framework. These clathrochelate molecules A and B in the X-rayed crystal of 2·0.75C_7_H_16_ have different types of hydrogen bonding. Two molecules 2 of type A with an encapsulated Fe1 ion and two molecules 2 of type B with a caged Fe2 cation form the H-bonded clathrochelate tetramers shown in Fig. 12 through their carboxylic and amide groups, respectively. These tetramers are connected into the H-bonded chains *via* the N–H⋯O interactions of an amide fragment of the clathrochelate molecule of type A and oxygen atom of a cage framework of the neighbouring macrobicyclic molecule of type B. As a result, in the X-rayed crystal 2·0.75C_7_H_16_, each of the macrobicyclic molecules 2 of type A is connected with four clathrochelate molecules 2 of type B, and each of the macrobicyclic molecules 2 of type B is H-bonded to four cage molecules 2 of type A.

**Table 1:** Main geometrical parameters of the fluoroboron-capped monoribbed-functionalized bis-α-benzildioximate arylsulfide iron(ii) clathrochelate molecules

Parameter	2		FeBd_2_((C_6_H_5_S)_2_Gm)(BF)_2_ [42]	FeBd_2_((*meta*-3-HOOC_6_H_4_S)_2_Gm)(BF)_2_ [11]	
	Type A	Type B		Type A	Type B
Fe–N (Å)	1.8754(6)–1.9286(4), av. 1.899	1.8845(5)–1.9155(6), av. 1.903	1.905(3)–1.920(3), av. 1.912	1.905(4)–1.918(4), av. 1.909	1.908(4)–1.926(4), av. 1.912
B–O (Å)	1.4622(5)–1.5043(4), av. 1.485	1.4454(5)–1.5222(4), av. 1.478	1.468(6)–1.503(5), av. 1.489	1.476(6)–1.497(6), av. 1.489	1.483(7)–1.503(6), av. 1.492
N–O (Å)	1.3602(6)–1.3708(6), av. 1.366	1.3606(5)–1.3990(5), av. 1.374	1.344(4)–1.373(4), av. 1.365	1.367(5)–1.377(5), av. 1.372	1.362(5)–1.371(5), av. 1.367
C=N (Å)	1.3119(3)–1.3302(3), av. 1.322	1.2851(3)–1.3403(3), av. 1.317	1.320(5)–1.332(5), av. 1.327	1.294(6)–1.319(6), av. 1.310	1.296(6)–1.318(5), av. 1.309
C–C (Å)	1.4332(6)–1.4470(6), av. 1.442	1.4449(3)–1.4513(5), av. 1.500	1.417(5)–1.448(6), av. 1.432	1.444(7)–1.461(6), av. 1.454	1.436(6)–1.457(6), av. 1.445
N=C–C=N (°)	11.43(1)–15.02(1), av.13.10	6.06(1)–12.29(1), av. 8.9	10.0(5)–14.9(5), av. 13.1	5.2(5)–11.6(6), av. 9.3	7.5(4)–13.0(5), av. 11.3
*φ* (°)	26.6	25.3	25.1	25.2	24.4
*α* (°)	78.9	78.0	79.0	78.4	78.6
*h* (Å)	2.31	2.30	2.34	2.33	2.34

### UV-vis and fluorescence spectra and UV-vis kinetic studies

The presence of highly intense metal-to-ligand charge transfer (MLCT) Fe d → Lπ* bands in the visible range is a characteristic of the UV-vis spectra of the tris-dioximate iron(ii) clathrochelates.^1,30^ The maxima of these bands are affected by the nature of the σ- and π-conjugated substituents in the chelating ribbed fragments of their quasiaromatic cage frameworks. In the case of the bis-α-benzildioximate iron(ii) clathrochelates, one absorption band has been observed from 400 to 550 nm with a maximum at approximately 470 nm.^16^ The presence of this broad and highly intense asymmetric band may result in a substantial decrease in a quantum yield or even in a complete quenching of the intrinsic fluorescence of the standard fluorescently active compounds, caused either by an excitation energy transfer or by reabsorption of their emission in a wide spectral range. Earlier, we succeeded in the preparation of a coumarin-functionalized iron(ii) clathrochelate; however, it was found to be a fluorescently silent compound. So, in the present study, we tested another fluorescently active group, a fluorescein residue, one of the most common and widely used fluorophore dyes, for target functionalization of the iron(ii) clathrochelates. This dye is known^20^ to possess relatively high molar extinction (approximately 8.8 × 10^4^ mol^−1^ L cm^−1^ at 499 nm in its basic aqueous solution^43^), with an excellent fluorescence quantum yield of 0.93 and good water solubility as well.

**Fig. 13:**
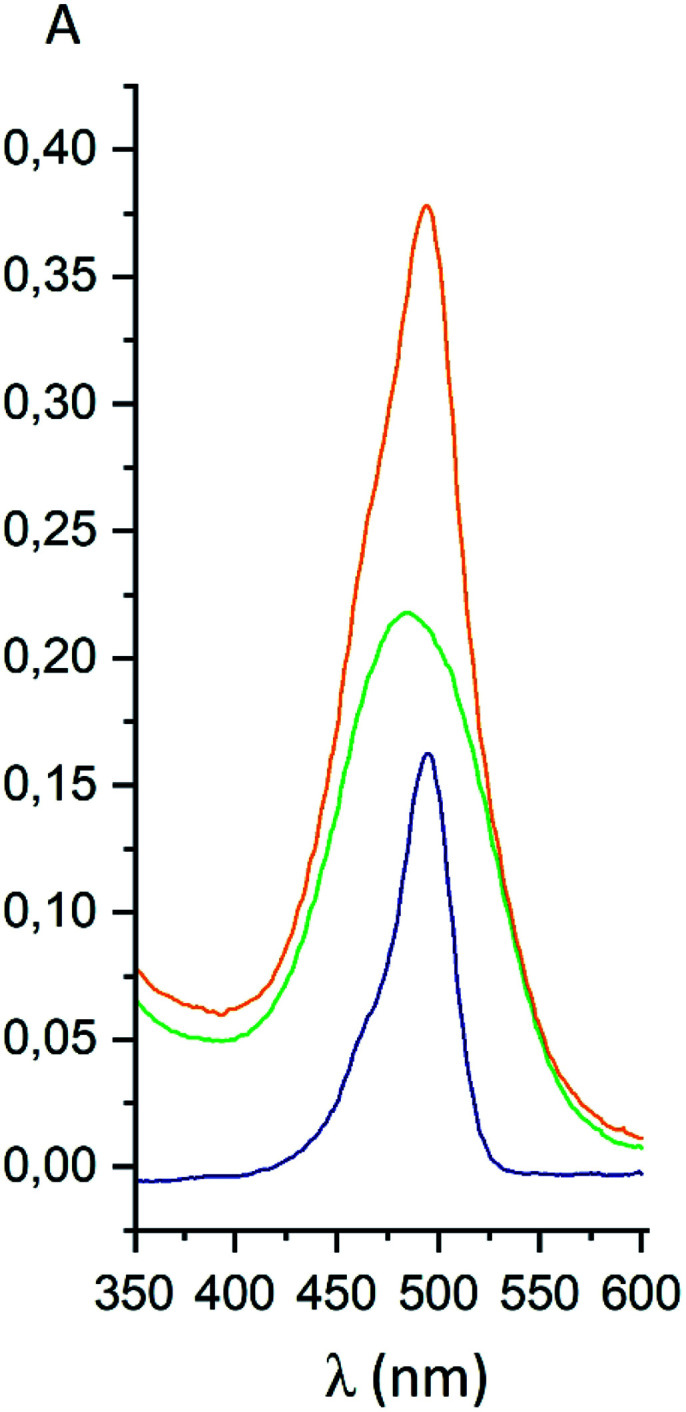
UV-vis spectra of fluorescein azide 5 (shown in blue), the tag-less clathrochelate 4 (shown in green) and their equimolar mixture (shown in orange).

**Fig. 14:**
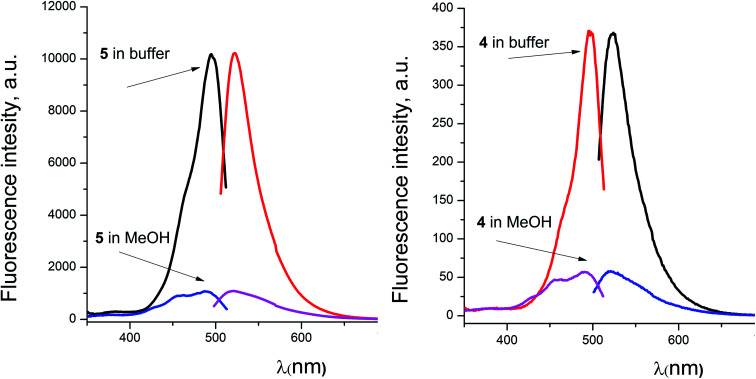
Excitation and fluorescence emission spectra of fluorescein azide 5 (left) and the fluorescein-tagged iron(ii) clathrochelate 4 (right) measured from their solutions in 0.05 M Tris–HCl aqueous buffer with pH 7.9 and in methanol.

For the fluorescein azide 5 and its tagged clathrochelate derivative 4, the UV-vis absorption and fluorescent spectra were measured from their 3 μM solutions both in 0.05 M Tris–HCl aqueous buffer with pH 7.9 and in methanol (Fig. 13); the obtained spectral data are collected in Table 2. Both these fluorescein derivatives were initially isolated in acidic media and, therefore, their dye fragment adopts its closed form (Fig. 9). As it can be seen from Fig. 14, compounds 4 and 5 possess different spectral characteristics in solvents of different polarity and H-donor ability. In their methanol solutions, a closed form of this dye fragment persists in both these molecules, while, in a slightly basic medium of the buffer solution, this labeling group adopts its open tautomeric form (Fig. 14), which possesses a higher fluorescence quantum yield. The calculated quantum yields for 5 and 4 are equal to 0.8 and 0.07, respectively, suggesting an 11-fold decrease in fluorescence quantum yield from the fluorescein dye 5 to its clathrochelate derivative 4. This effect can be explained by an excitation energy transfer from a terminal fluorescein group of labeled molecule 4 to its quasiaromatic highly π-conjugated clathrochelate framework.

**Table 2:** Fluorescence characteristics of compounds 4 and 5 measured in two different media

Compound	Tris–HCl aqueous buffer			Methanol		
	*λ*_ex_ (nm)	*λ*_em_ (nm)	*I* (a.u.)	*λ*_ex_ (nm)	*λ*_em_ (nm)	*I* (a.u.)
4	497	523	368	490	521	57
5	495	522	10181	487	523	1045

**Fig. 15:**
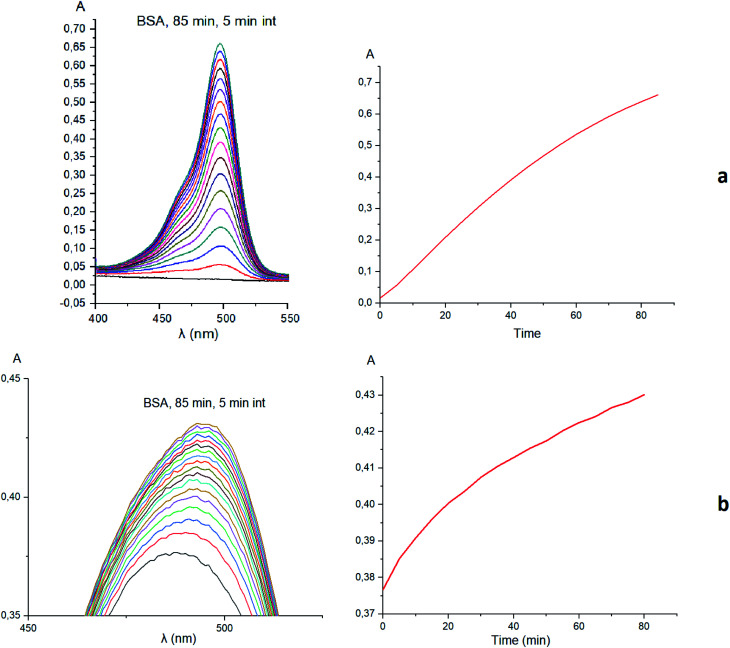
Kinetic UV-vis experiments and the corresponding plots illustrating the supramolecular host–guest binding of fluorescein azide 3 (a) and clathrochelate complex 1 (b) as the guests to BSA macromolecule as a host.

**Fig. 16:**
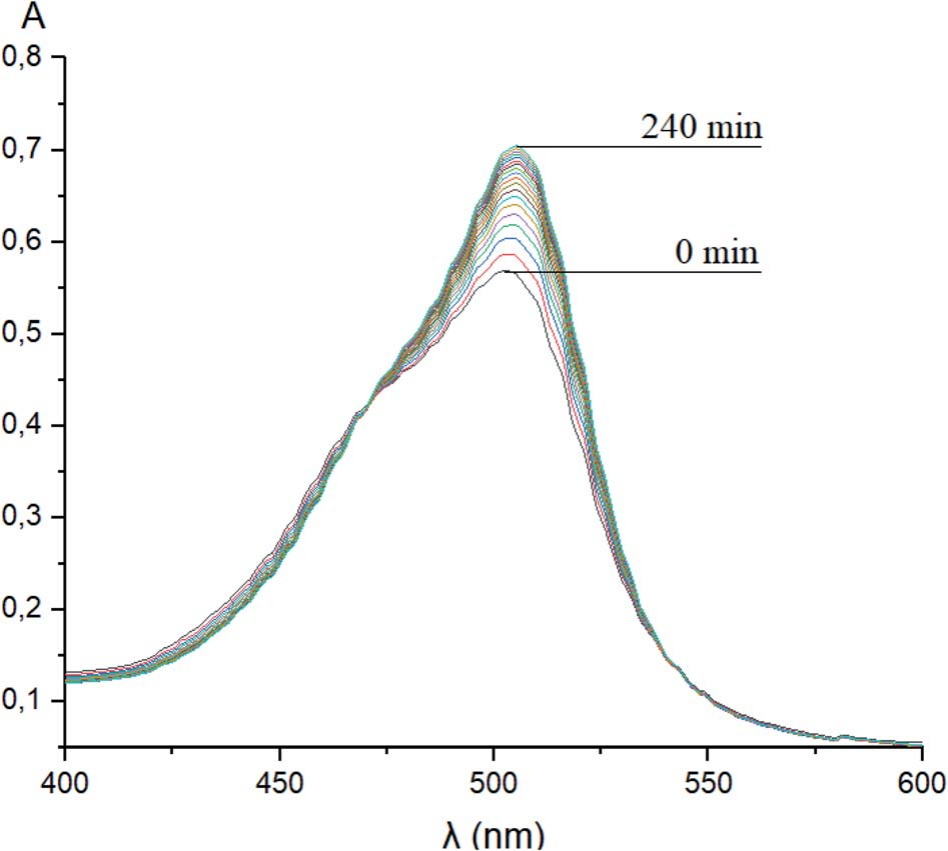
Changes over time of the solution UV-vis spectrum of complex 4 in the presence of BSA; 0.05 M Tris–HCl aqueous buffer with pH 7.9 was used as a solvent.

Taking into account the well-known^44,45^ esterase-like activity of serum albumins, we performed a study of kinetics of a hydrolysis of the acetyl groups of molecules 1 and 3 in the presence of one equivalent of BSA. Both molecules underwent this reaction, leading to a transformation of their fluorescein-containing closed fragment into its opened tautomeric form, as well as causing an increase in the intensities of their absorptions. Similar optical outputs were observed for both compounds 1 and 3 (Fig. 15), thus suggesting the absence of their (as the guests) strong supramolecular host–guest interactions with the binding centres of BSA macromolecule as a host. On the other hand, the study of a chemical stability of complex 4 showed an increase in the intensity of its characteristic visible absorption band over time in the presence of one equivalent of BSA (Fig. 16); this spectral effect was not observed in the absence of this transport protein. Therefore, it could be caused by formation of a supramolecular BSA–clathrochelate 4 assembly in the Tris–HCl aqueous buffer solution. Due to the very high halfwidth of the abovementioned visible absorption band, we were not able to assign the components that appeared because of the formation of this non-covalent associate. On the other hand, its dissociation caused an increase in the optical density of the obtained solution. This also evidenced the formation of a supramolecular host–guest assembly between the hosting BSA macromolecule and the clathrochelate guest 4. Without BSA, no significant change in the measured spectrum was observed for the same period of time. The UV-vis spectrum of the solution of complex 4 in 0.05 M Tris–HCl aqueous buffer with pH 7.9 was measured after 7 days of being kept at room temperature and a 3.5% decrease in its maximal adsorption was observed. We explained this effect by the slow precipitation (agglomeration) of this complex in the given buffer solution while retaining its cage structure.

### Clathrochelate-based ICD spectra

**Fig. 17:**
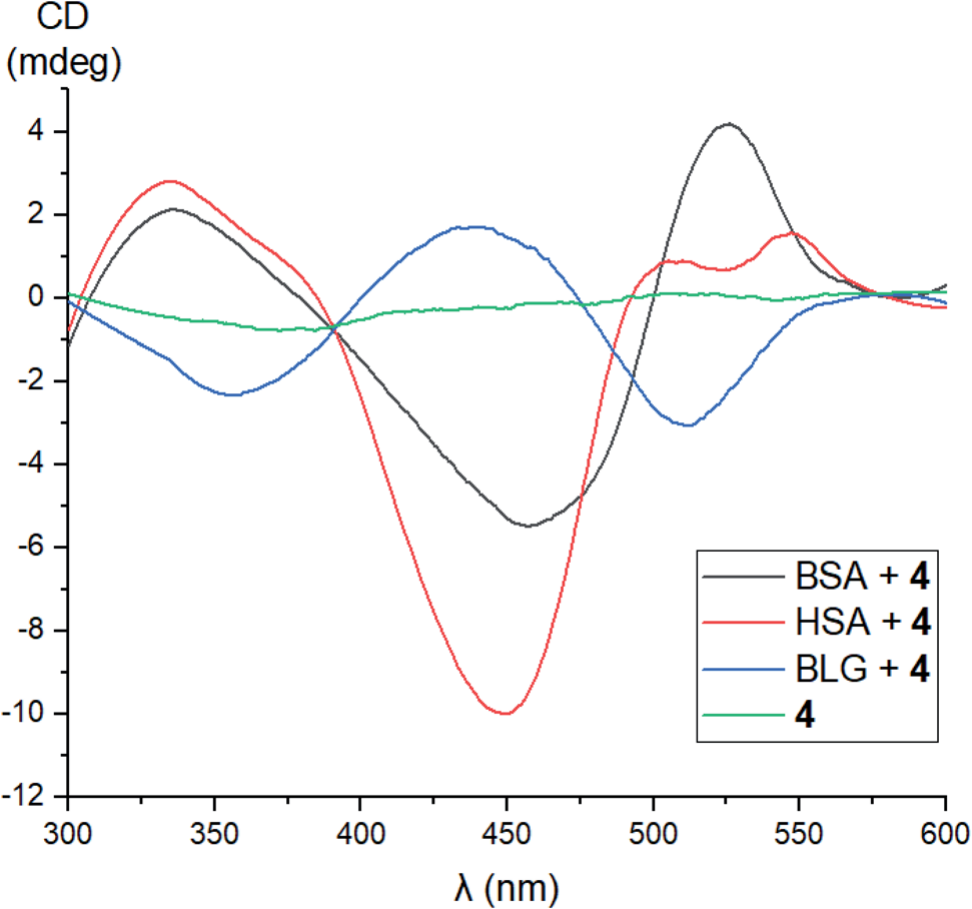
CD spectra of the supramolecular assemblies of proteins HSA, BSA, BLG and LYZ as the hosts with iron(ii) clathrochelate 4 as the guest, measured in 0.05 M Tris–HCl aqueous buffer with pH 7.9 at 25 °C.

No signals were observed in the CD spectra of the initial acetylated compounds 1, 3, and 5. On the other hand, because of a lipase activity of globular proteins, the CD silence of acetylated complex 1 in the presence of these proteins and the appearance of CD signals for its deacetylated derivative 4 under the same reaction conditions suggest their supramolecular binding as clathrochelate guests to the different binding sites of their macromolecules. Indeed, in the case of macrobicyclic complex 4, the addition of BSA or HSA caused the appearance of strong clathrochelate-based ICD outputs with two positive (maxima at approximately 350 and 520 nm) and one negative (maximum at approximately 450 nm) bands possessing various intensities (Fig. 17). The shapes of these ICD spectra are very similar to those observed earlier^46^ for their untagged clathrochelate analogs. Host–guest binding of molecule 4 to the BLG macromolecule caused the appearance of two negative (maxima at approximately 350 and 520 nm) and one positive (maximum at approximately 450 nm) bands possessing similar intensities. In the case of LYZ as a hosting macromolecule, the corresponding CD spectra were not informative because of the formation of an insoluble LYZ–clathrochelate 4 supramolecular assembly.

### Fluorescence emission anisotropy data

**Fig. 18:**
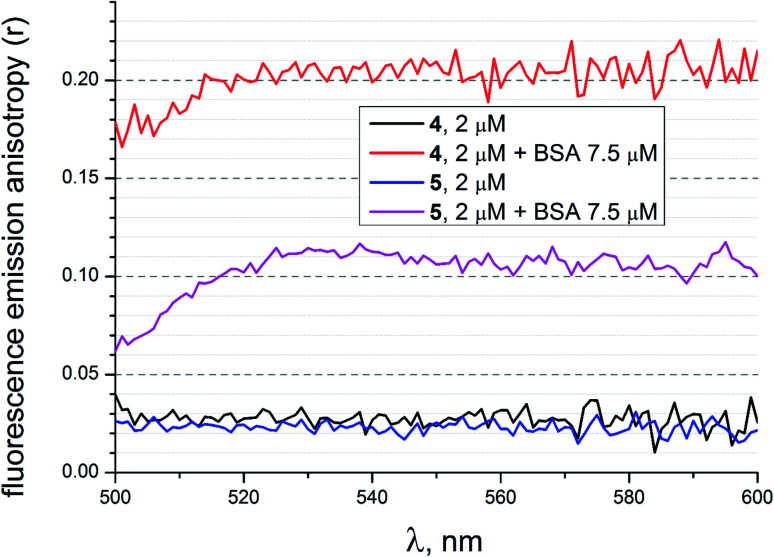
Fluorescence emission anisotropy spectra of compounds 4 and 5 with excitation at 475 nm alone and those in the presence of BSA, measured in 50 mM Tris–HCl aqueous buffer with pH 7.9.

To evaluate additional evidence of the supramolecular binding of a given guest molecule to the hosting BSA macromolecule, we performed fluorescence emission anisotropy studies of compounds 4 and 5 (2 μM) in the absence and presence of BSA (7.5 μM) in 50 mM Tris–HCl aqueous buffer with pH 7.9, using an excitation at 475 nm (Fig. 18). As it can be seen from Fig. 18, the presence of BSA led to a high increase in anisotropy in the cases of both the fluorescein-tagged clathrochelate 4 and the fluorescein-based compound 5. This suggests that their molecules as the guests form stable supramolecular assemblies with the BSA macromolecule. The observed decrease in fluorescence anisotropy for the solutions of 4 and 5 in the presence of BSA at wavelengths lower than 520 nm can be explained by the greater contribution of these initial non-bonded molecules (*i.e.* those which are not included in the formation of the host–guest assemblies) to their fluorescence spectra in the corresponding shorter-wavelength spectral range.

### Molecular imagining of fluorescein-tagged iron(ii) clathrochelate in cancer cells

**Fig. 19:**
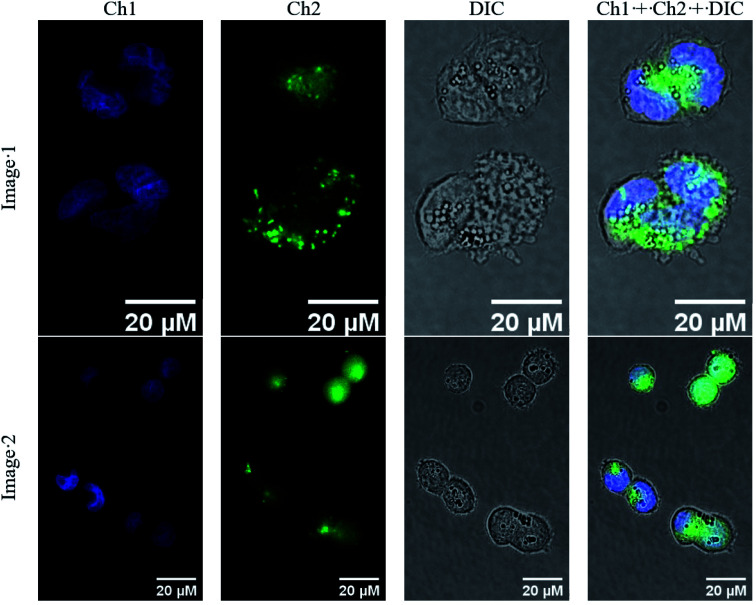
Accumulation of fluorescein-tagged iron(ii) clathrochelate 4 in A2780 cancer cells. Cells were treated with 4 for 4 h and subsequently stained with Hoechst 33342. Fluorescence images were taken in addition to brightfield images (DIC). Fluorescence channels: Channel 1 (Ch1, blue), *λ*_ex_: 365 nm; *λ*_em_: 445/50 nm (detection of Hoechst in nuclei); Channel 2 (Ch2, green): *λ*_ex_: 470/40 nm; *λ*_em_: 525/50 nm (detection of compound 4). Images were processed and merged (Ch1 + Ch2 + DIC) using ImageJ.

The obtained novel fluorescently active iron(ii) clathrochelate 4 was additionally characterized in a cellular context. For this purpose, A2780 cancer cells were incubated with 4 for 4 h and their nuclei were stained using Hoechst 33342; the subcellular localization of this tagged cage complex was visualized using fluorescence microscopy. Despite the observed fluorescence quenching behaviour of the quasiaromatic metal clathrochelates (see above), a reasonable fluorescent signal of the fluorescein group of the corresponding functionalized cage molecule is expected to be detectable because of both its high quantum yield and its high emission intensity. Indeed, an intense optical signal was detected for complex 4 in the 38 blue channel (Fig. 19, shown in green) which is not colocalized with the corresponding Hoechst signal in the 49 DAPI channel (Fig. 10, shown in blue). This suggests that fluorescein-tagged clathrochelate 4 is efficiently taken up by cancer cells, where it is evenly distributed in the cytosol. This fluorescein-tagged iron(ii) cage complex does not seem to enter the nucleus and no accumulation in any other organelle is apparent. Unfortunately, no toxicity was observed for tagged clathrochelate 4.

## Conclusions

We elaborated an efficient synthetic strategy to obtain the first fluorescein-tagged iron(ii) clathrochelates functionalized with a terminal fluorophore group and studied their chemical stability in various media, their self-assembly with globular proteins, and their localization in cancer cells.

In the X-rayed crystal of clathrochelate precursor 2, molecules of two types, A and B, were found.

It was shown that fluorescein azide and its clathrochelate derivative possess different spectral characteristics: an 11-fold decrease in a fluorescence quantum yield is observed between a given fluorescein-based dye and this macrobicyclic complex.

A study of kinetics of a hydrolysis of the acetyl groups of acetylated fluorescein azide and its clathrochelate derivative in the presence of one equivalent of BSA evidenced that there are no strong supramolecular host–guest interactions between the BSA macromolecule and these tested compounds. At the same time, during the study of achemical stability of the deacetylated clathrochelate, the formation of a supramolecular 1 : 1 BSA–clathrochelate assembly was detected. Moreover, the addition of BSA or HSA to its solution caused the appearance of strong clathrochelate-based ICD outputs with two positive and one negative bands possessing different intensities. The fluorescence emission anisotropy studies also evidenced the supramolecular binding of fluorescein-tagged iron(ii) clathrochelate to the BSA macromolecule, leading to a high increase in such anisotropy.

The cellular uptake of the fluorescein-tagged molecules was visualized using fluorescence microscopy which showed that the compound was mainly distributed in the cytosol without entering into the nucleus or accumulating in any other organelle.

Thus, the designed fluorescently active cage complexes seem to be prospective molecular probes for studies of subcellular localization. As a result, the use of the obtained fluorescent clathrochelates in further experiments can give new insights into the mechanism of bioactivity of clathrochelate-based drug candidates, which are promising therapeutically active compounds to fight viral, neurodegenerative, and cancerous diseases.

## Author contributions

The manuscript was written through the contributions of all authors. All authors read and approved its final version; they all contributed equally.

## Conflicts of interest

There are no conflicts of interest to declare.

## Supplementary Material

RA-011-D0RA10502C-s001
